# Structure Optimization of a Fe–Mn–Pd Alloy by Equal-Channel Angular Pressing for Biomedical Use

**DOI:** 10.3390/ma16010045

**Published:** 2022-12-21

**Authors:** Olga Rybalchenko, Natalia Anisimova, Natalia Martynenko, Georgy Rybalchenko, Mikhail Kiselevskiy, Natalia Tabachkova, Igor Shchetinin, Arseniy Raab, Sergey Dobatkin

**Affiliations:** 1 A.A. Baikov Institute of Metallurgy and Materials Science of the Russian Academy of Sciences, 119334 Moscow, Russia; 2 N.N. Blokhin National Medical Research Center of Oncology (N.N. Blokhin NMRCO) of the Ministry of Health of the Russian Federation, 115478 Moscow, Russia; 3 Center for Biomedical Engineering, National University of Science and Technology “MISIS”, 119049 Moscow, Russia; 4 P.N. Lebedev Physical Institute of the Russian Academy of Sciences, 119991 Moscow, Russia; 5 A.M. Prokhorov General Physics Instituteof the Russian Academy of Sciences, 119991 Moscow, Russia; 6 Department of Physical Materials Science, National University of Science and Technology “MISIS”, 119049 Moscow, Russia; 7 Institute of Physics of Advanced Materials, Ufa University of Science and Technology, 450000 Ufa, Russia; 8 Department of Metal Science and Physics of Strength, National University of Science and Technology “MISIS”, 119049 Moscow, Russia

**Keywords:** equal-channel angular pressing, Fe–Mn–Pd alloys, phase transformation, corrosion rate, mechanical properties, biocompatibility

## Abstract

In this work, a Fe–Mn–Pd alloy was produced by methods of equal channel angular pressing (ECAP) in order to obtain an alloy with a high rate of degradation for the development of biodegradable devices. Special efforts were made to the obtaining of an ultrafine-grained structure of alloys in a fully austenitic state at temperatures of 300 °C and 450 °C. Further investigation of its effect on the corrosion rate and mechanical properties was carried out. The formation of an austenitic structure with structural element sizes of 100–250 nm after deformation was confirmed by X-ray diffraction analysis. ECAP proved to be the reason for a significant increase in strength with maximum σ_UTS_ = 1669 MPa and σ_YS_ = 1577 MPa while maintaining satisfactory plasticity. The alloy degradation rate was investigated using the potentiodynamic polarization analysis. The corrosion rate of the alloy after ECAP (~1 mm/y) is higher than that of the coarse-grained state and significantly higher than that of annealed iron (~0.2 mm/y). ECAP in both modes did not impair the biocompatibility of the Fe–Mn–Pd alloy and the colonization of the sample surface by cells.

## 1. Introduction

Recently, significant efforts have been paid to the development of bioresorbable alloys for temporary biomedical devices [1,2,3]. Among such metallic materials, alloys based on Mg, Zn and Fe are potentially attractive [1,2,3,4,5]. Fe-based alloys have excellent mechanical characteristics, which allows them to be used as a material for osteosynthesis [3,4,6], as well as in cardiovascular surgery [7,8,9,10,11,12]. However, their low rate of biodegradation is a significant disadvantage that negatively affects long-term biocompatibility. In order to solve this problem, alloying is usually used [12,13]. Mn is one of the few biocompatible alloying elements that results in a reduction in the standard electrode potential of the iron matrix and a significant increase in corrosion susceptibility [5,10,14,15]. Nevertheless, in [15], the addition of manganese in the amount of 35 wt. % did not increase the corrosion rate to the level required to create temporary medical implants.

In [16], a strategy was developed to achieve an increased rate of degradation of iron-based alloys. The idea is to increase the corrosion rate not only by the addition of more electronegative elements but also by alloying with more inert elements due to the possible precipitation of a dispersed intermetallic phase (IMP) during subsequent annealing. In this case, IMP is acting as a cathode, creating a micro-galvanic couple with the iron matrix and causing accelerated uniform corrosion. The authors demonstrated the effectiveness of their strategy on the Fe–10Mn–1Pd alloy, which showed a significantly higher degradation rate and attractive mechanical characteristics [16]. A similar combination of an increased corrosion rate, high strength and ductility was demonstrated both on martensitic Fe–Mn–Pd alloys [17] and on austenitic Fe–Mn–C–Pd alloys [18]. However, the developed alloys [16,17] are not suitable for medical use due to the presence of a ferromagnetic α’-phase. Unfortunately, the high magnetic susceptibility of such iron-based alloys is another limitation of their clinical use. The presence of a paramagnetic state (γ-austenite, ε-martensite) will allow the use of magnetic resonance imaging (MRI) for accurate estimation of the condition of the implanted device [14].

In this work, by using the basic thesis of the developed strategy [16], together with metallurgical methods for increasing the degradation rate, methods of severe plastic deformation (SPD) were used. One of the important tasks of this work was to investigate the possibility of increasing the degradation rate of Fe–Mn–Pd alloys, mainly by creating an austenitic ultrafine-grained (UFG) structure using the equal channel angular pressing (ECAP) method. The ECAP technique was used for this task since it can help to develop bulk samples suitable for use in various medical devices. Previously, a good biological response of cells to the nano- and ultrafine-grained metal structure was noted [19,20]. For example, in [19], it was shown that the interaction of various cells with nanocrystalline iron obtained by the ECAP method stimulates better proliferation of fibroblasts compared to the coarse-grained state, good ability for endothelization, and at the same time, effectively inhibits the viability of vascular smooth muscle cells. In [19], Nie et al. concluded that nanocrystalline pure iron has good biocompatibility in vitro. In [20], improved proliferation, good attachment and growth of pre-osteoblasts cell line MC3T3-E1 was noted on samples of stainless steel with nano- and UFG structures.

It is known that metals in the ultrafine-grained state are characterized by a significant volume of structural element boundaries and an increased density of dislocations [21,22,23]. A high density of structural defects in nanocrystalline materials should lead to a significant increase in stored energy and improve reactivity, which in turn can increase the susceptibility of the alloy to corrosion [24]. However, this structural state has a more complex effect on corrosion propensity. The corrosion behavior of UFG alloys will depend on the interaction between the material and the medium [25,26,27,28]. For example, it was found in [19] that the obtained UFG structure after ECAP does not increase the rate of degradation of pure iron but rather reduces it. In the earlier work [29], ECAP of Fe-Mn-C alloys led to an increase in the corrosion rate. It was shown that an increase in the tendency to biodegradation is due not only to the refinement of the structure but also to a change in the phase composition of the material (precipitation of α-Mn) during deformation [29]. In this work, it was assumed that ECAP at elevated temperatures should provide precipitation of the intermetallic phase (IMP) rich in Pd, which would accelerate the process of biodegradation by the creation of UFG structures, and by electrochemical corrosion due to more uniformly dispersed distributed IMP particles. It is expected that the cumulative effect of alloying and ECAP will lead to the development of a Fe–Mn–Pd alloy that meets the requirements for the manufacturing of medical implants in terms of a set of functional (mechanical, chemical and biological) characteristics.

## 2. Materials and Methods

The Fe–Mn–Pd alloy was obtained in the Leybold Gereus L200DI vacuum arc remelting furnace (VAR)(Leybold, Cologne, Germany) from commercially pure iron, electrolytic manganese (≈ 99.8% Mn) and Fe−3% Pd master alloy. The billet was remelted up to 6 times. The obtained sample in the form of a disk with a diameter of 120 mm was forged at a temperature of 1100 °C to obtain rods with a diameter of 12 mm. Subsequent homogenization annealing was carried out at a temperature of 1250 °C for 12 h in quartz tubes in a vacuum. The elemental analysis of the alloy was determined by Wavelength Dispersive X-ray Fluorescence (WDXRF) spectrometer S8 Tiger (Bruker AXS, Karlsruhe, Germany). The measurements were carried out in a vacuum according to the standard procedure using the Quantum-Express^TM^ software (Bruker, Karlsruhe, Germany). The carbon concentration was determined using a LECO carbon determinator (LECO, Vouersweg, Netherlands). Table 1 summarizes the alloy compositions.

ECAP was carried using a die with intersecting at 120° channels via route Bc with a graphite lubricant used to minimize frictional effects at the die walls [30]. Samples for ECAP 60 mm long were turned to a diameter of 10 mm. ECAP was performed at two temperatures, 300 °C and 450 °C. This made it possible to deform the high-strength alloy up to four ECAP passes. The equivalent cumulative strain applied to the sample, calculated for a given die geometry, was 3.6 [31,32].

The microstructure of the sample in the coarse-grained state after annealing was investigated using a JSM−7001F (JEOL; Tokyo, Japan) scanning electron microscope. The microstructure after ECAP was studied by JEM−2100 transmission electron microscope (TEM) (JEOL; Tokyo, Japan) with 200 kV electron beam energy. Thin foils for TEM were mechanically ground to 0.09 mm and thinned to perforation by a TenuPol−5 Struers unit (Struers; Copenhagen, Denmark) with a polishing electrolyte precipitated of a mixture of perchloric acid, ethanol, butoxyethanol and distilled water at 20 V. The phase composition was investigated by X-ray diffraction (XRD). It was performed by a Rigaku Ultima IV diffractometer (Rigaku; Tokyo, Japan) with CoK_α_ radiation (wavelength: λ = 1.7902 Å).

Uniaxial tensile tests were carried out on an INSTRON 3380 machine (Instron, Norwood, MA, USA) with a load of 100 kN at a crosshead travel speed of 1 mm/min at room temperature. Tensile samples were cut from billet after ECAP on an electric spark machine with a working part length of 5.75 mm and a cross-section of 2 × 1 mm. Samples for studying mechanical characteristics were cut in a direction parallel to the direction of deformation. The samples were mechanically polished using SiC paper of different grain sizes and diamond paste. Microhardness measurements were carried out using a 402 MVD Wolpert Wilson^®^ micro-hardness tester. Vickers hardness under a load of 0.1 kgf with a holding time of 15 s was measured. For each group, an average of at least ten measurements were taken.

The corrosion resistance was evaluated by the electrochemical measurements using an SP−300 (Bio-Logic SAS; Seyssinet-Pariset, France) potentiostat. A flat PAR cell 350 mL (Ametek Instruments, Oak Ridge, TN, USA) with a “three-electrode configuration” was used. A sample with an area of 0.8 cm^2^ was used as a working electrode. The reference was a saturated calomel electrode (SCE). A platinum grid was used as a counter electrode. The distance between the working electrode and the tip of the bridge tube was kept constant in order to minimize the IR drop in the solution. The environment for the corrosion experiments was the physiological saline solution (0.9% NaCl dissolved in distilled water) with a pH value of 7.4 at room temperature. The sample for the working electrode was ground with SiC paper with decreasing grain size up to P2000, degreased with ethanol and rinsed with distilled water. The scanning by potentiodynamic polarization (PDP) was carried out at a rate of 1 mV/s. The time for determining the open circuit potential was 20 min. The corrosion potential, corrosion current density and corrosion rate (CR) were obtained using the EC-Lab Software (BioLogic, Seyssinet-Pariset, France).

In order to study bioactivity in vitro, samples in the form of a quarter of a disk 10 mm in diameter and about 1 mm thick were used. Samples were mechanically polished using SiC paper of different grain sizes up to P2000, washed with deionized water, immersed for 4 h in 70% ethanol and then dried under sterile conditions. For each test in the initial state and after ECAP at temperatures of 450 °C and 300 °C, nine samples of the Fe–Mn–Pd alloy were used.

In order to assess the biocompatibility of the alloy, their effect on hemolysis and cell viability was evaluated according to the methods described earlier [33]. The ability of alloy samples to induce hemolysis was studied by incubation in 2 mL of sterile physiological sodium chloride solution (PanEco, Russia) with red blood cells of C57 BL/6 mice (6,860,000 cells in 1 mL) for 4 h and 24 h at 37 °C in an atmosphere with 5% carbon dioxide. A suspension of cells incubated under the same conditions but without alloy samples were used as a control. Before the start of the experiment, the baseline was determined by measuring the absorption of the supernatant in the control at 540 nm versus 690 nm using a Spark plate reader (Tecan, USA). At the end of the incubation, the absorption of the supernatant was evaluated, and the level of hemolysis induced by the alloy and in control was calculated relative to the baseline in percent.

In order to assess the effect of alloys on maintaining cell viability, samples were incubated in a complete medium based on RPMI−1640 medium (PanEco, Russia) supplemented with 10% fetal serum (Sigma), penicillin (100 U/mL) and 2 mM glutamine (both–PanEco, Russia) with blood mononuclear leukocytes of C57 BL/6 mice (640,000 cells per 1 mL) for 24 h at 37 °C in an atmosphere with 5% carbon dioxide. As a control, leukocytes were incubated without alloy samples under the same conditions. Cell viability was investigated in the lactate dehydrogenase (LDH) activity assay using the Pierce™ LDH Cytotoxicity Assay Kit (Thermo Scientific, USA) according to the manufacturer’s instructions. At the end of incubation, the absorption of the medium after cell lysis was assessed at 492 nm (A 492) using a Spark plate reader (Tecan, USA), and the LDH activity of cells at the end of incubation was calculated relative to the baseline LDH activity in percent. The baseline was determined in the control before the incubation of cells with alloy samples.

In order to study the ability of the alloy to stimulate the colonization of the surface of samples by cells, multipotent mesenchymal stromal cells (MMSCs) of mice (collection of cell lines of N.N. Blokhin NMRC) were used. An amount of 20 μl of MMSC in complete RPMI−1640 medium was seeded to the upper surface of the alloy samples (1,200,000 cells in 1 mL) and kept for 30 min at 37 °C in an atmosphere with 5% carbon dioxide; additionally, 2 mL of a fresh portion of the medium was added and incubated under the same conditions for 4 days. In the control, the cells were incubated at the bottom of the well of a 24-well plate (Nunc, USA) under similar conditions. The results were recorded taking into account the LDH activity (A492) of cells on the surface of the alloy samples in comparison with the control, as described above.

The experiments and procedures with cells and animals were assessed and approved by the Ethical Committee of the N.N. Blokhin NMRCO (protocol #AAAAA-A19–119). Each parameter was measured in triplets. The results of statistical analysis were presented as a mean ± standard deviation (Mean ± SD). Comparisons between the two groups were made using Student’s t-test. Differences were considered statistically significant at *p* < 0.05.

## 3. Results

**Figure 1:**
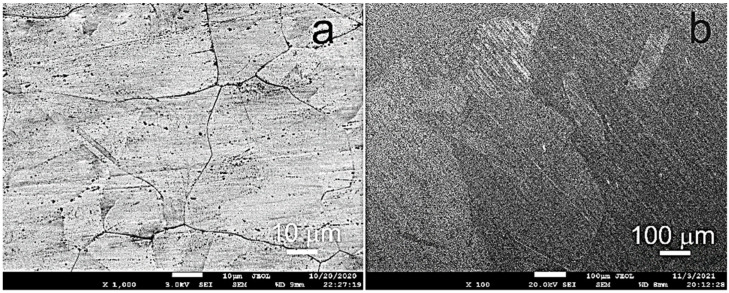
SEM images of the Fe–Mn–Pd alloy after forging (**a**) and annealing (**b**).

Figure 1 shows the initial structure of the alloy in the forged (Figure 1a) and annealed (Figure 1b) states. The grain size of Fe–Mn–Pd alloy after forging was 29 ± 2 μm. In the state after 12 h of annealing, a coarse-grained structure of the alloy with a grain size in the range of 230–750 μm and annealing twins of about 80 μm was revealed.

**Figure 2:**
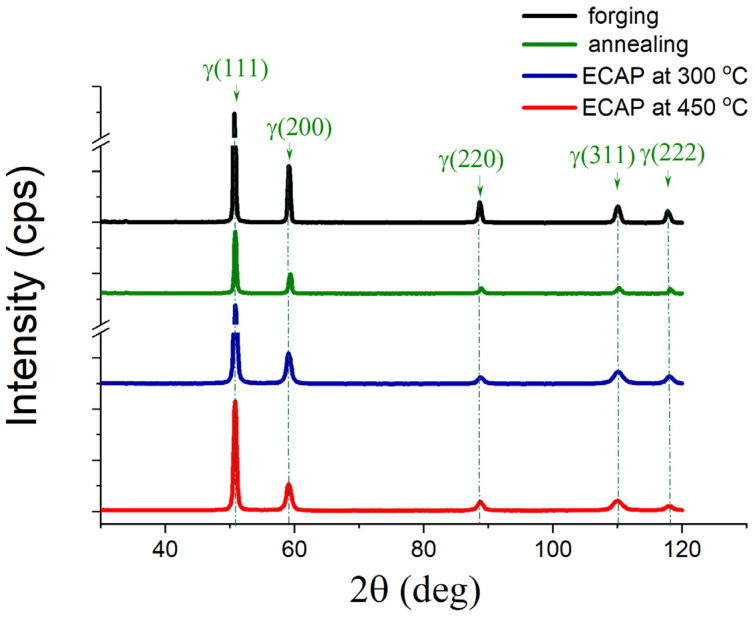
XRD patterns of the Fe–Mn–Pd alloy after various processing.

Usually, during the deformation of Fe-Mn alloys, an *γ → ε* martensitic transformation occurs [34,35]. However, during four ECAP passes at a deformation temperature of 300 °C and 450 °C, the martensitic *γ → ε* transformation was not realized. X-ray phase analysis showed a completely austenitic state in the Fe–Mn–Pd alloy both in the initial (forged and annealed) and deformed states after ECAP at temperatures of 300 °C and 450 °C (Figure 2, Table 2). The dislocation density was determined by recalculating the microstrains obtained by complete fitting of the diffraction profile by the Rietveld method.

The structure of the alloy after ECAP at 450 °C has a five times lower dislocation density compared to the alloy samples after ECAP at 300 °C, which is explained by a higher deformation temperature at the same equivalent cumulative strain.

**Figure 3:**
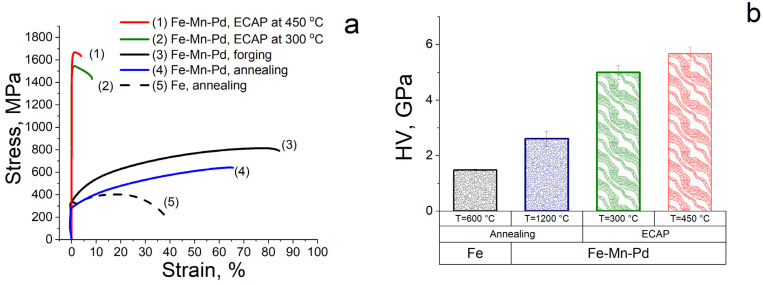
Tensile strain diagram (**a**) and microhardness (HV) of Fe–Mn–Pd alloy (**b**) after various processing (annealed Fe for comparison).

Mechanical properties were evaluated using tensile test results (Figure 3a, Table 3) and Vickers microhardness measurements (Figure 3b). The structure of the Fe–30 Mn–Pd alloy, obtained by the ECAP method at a temperature of 300 °C, increases its strength characteristics by a factor of 2.4 for ultimate tensile strength (σ*_UTS_* = 1545 MPa) and by a factor of 5.3 for yield strength (σ_YS_ = 1488 MPa) compared to the initial state (σ*_UTS_* = 643 MPa, σ_YS_ = 280 MPa). At the same time, the relative elongation after ECAP at 300 °C decreases from 65.5% to 8.5%, remaining at an acceptable level. With the increase in the ECAP temperature up to 450 °C at the same number of passes (applied deformation), the ultimate tensile strength of the Fe–30 Mn–Pd alloy increases up to σ*_UTS_* = 1669 MPa, and the total elongation decreases to ε= 4%. Higher strength characteristics and low ductility indicate quite noticeable phase changes that occur during the deformation process. Thus, despite the austenitic state of the alloy revealed by XRD after ECAP at deformation temperatures of 300 °C and 450 °C, mechanical tests show the possible presence of strengthening particles in the deformed structure of the samples.

The microhardness of the Fe–Mn–Pd alloy after ECAP at deformation temperatures of 300 °C and 450 °C increases by a factor of 1.9–2.2 compared to the initial annealed state (2.6 ± 0.3 GPa) to HV = 5.0 ± 0.2 GPa and up to HV = 5.7 ± 0.2 GPa, respectively (Figure 3b). Higher values of microhardness after ECAP at a temperature of 450 °C also indicate the precipitation of the second phase during deformation at a given temperature. This phase, due to a small amount of dispersion, could not be determined by X-ray phase analysis.

**Figure 4:**
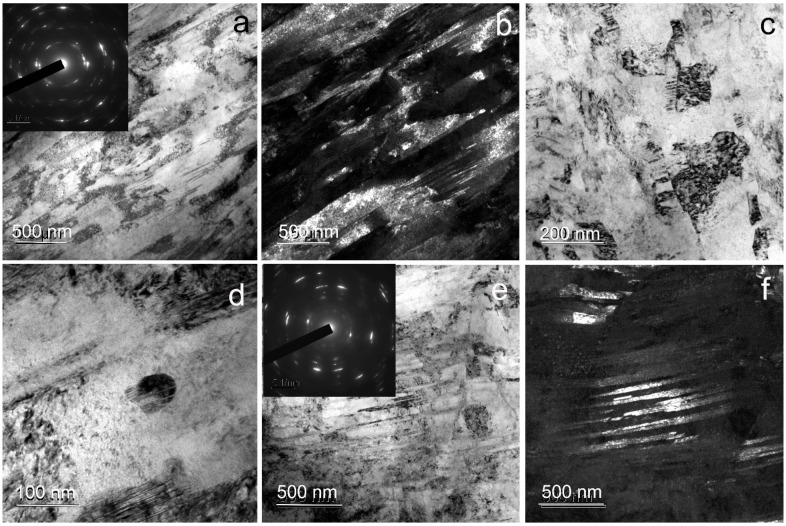
TEM images of Fe–Mn–Pd alloy after ECAP at 300 °C: (**a**,**c**,**d**) BF image of subgrain area with SAED pattern can be seen in the inset (**a**); (**b**) DF image of subgrain area; (**e**) BF image of twins area with SAED pattern can be seen in the inset; (**f**) DF image of twins area.

Transmission electron microscopy of Fe–Mn–Pd alloy samples after ECAP at 300 °C revealed the formation of an ultrafine-grained structure (Figure 4). According to the ring electron diffraction pattern, the structure is mainly subgrain. Its formation occurs due to the cutting of shear bands with a thickness of 84 ± 10 nm by thick dislocation bridges (Figure 4a–c), as well as due to twinning in austenite. Figure 4 shows regions with twins of 30 ± 3 nm thick (Figure 4e,f). Sometimes particles up to 40 nm in size were observed in the structure (Figure 4d).

**Figure 5:**
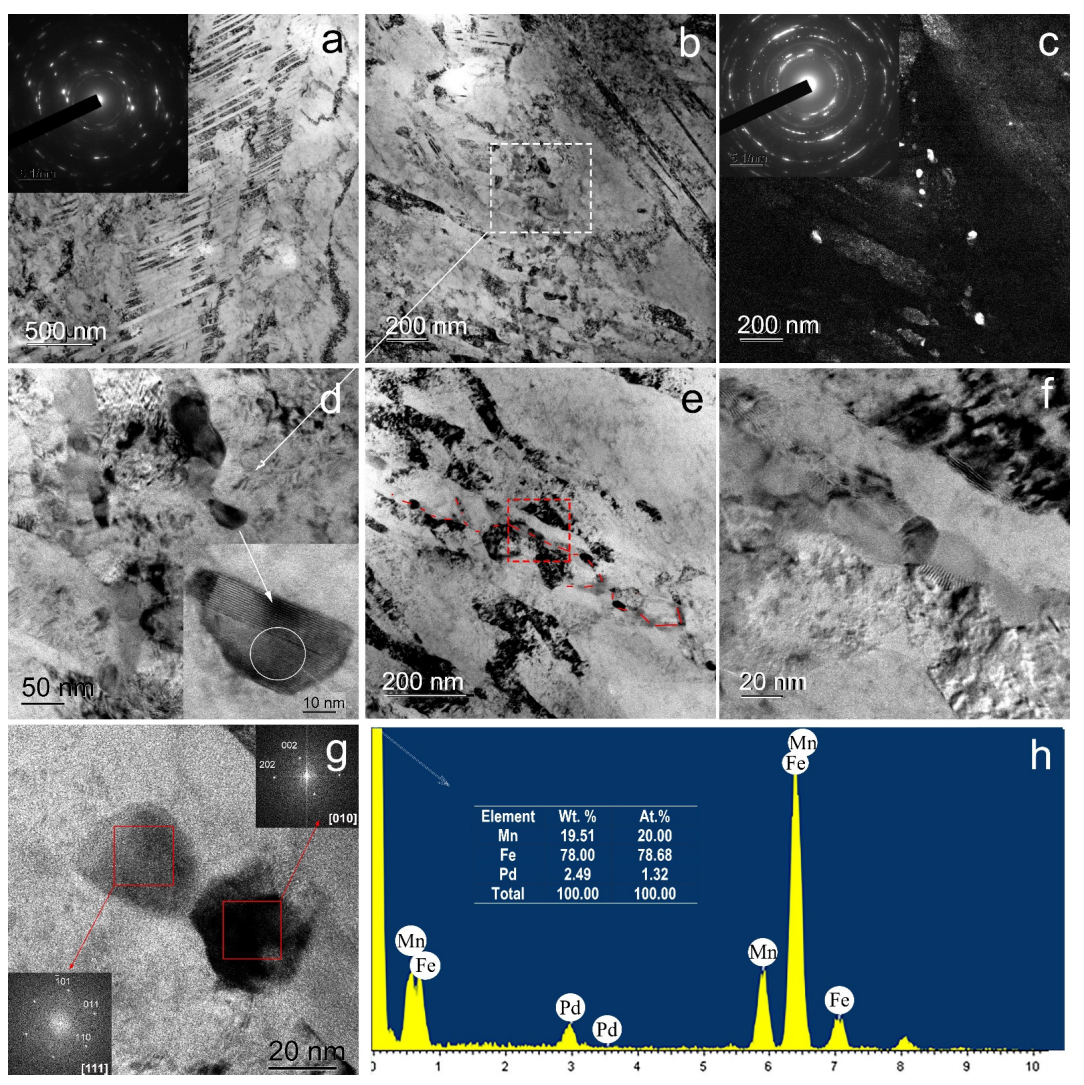
TEM images of Fe–Mn–Pd alloy after ECAP at 450 °C: (**a**) BF image of twins area with SAED pattern can be seen in the inset; (**b**,**d**–**f**) BF image of subgrain area; (**c**) DF image of subgrain area with SAED pattern can be seen in the inset; (**g**) HRTEM of Mn_2_Pd_3_ particle with FFT pattern from Mn_2_Pd_3_ particle can be seen in the inset; (**h**) and TEM-EDS point analysis result of the particle.

During ECAP at 450 °C, two types of UFG structures are also formed in shear bands 211 ± 35 nm thick (Figure 5). Some areas are twins in austenite 24 ± 1 nm thick crossing shear bands (Figure 5a).

Other areas where a subgrain structure is formed (Figure 5b–g) are thick dislocation walls of a cellular structure and shear bands. At the same time, in the places of formation of a subgrain structure in the thick boundaries of subgrains, the formation of coherent and incoherent particles with a size of 26 ± 1 nm occurs (Figure 5d–g). According to the FFT (Fast Fourier Transform) pattern from an individual particle shown in Figure 5g, the interplanar spacing of the resulting phase corresponds to Mn_2_Pd_3_ particles.

The TEM-EDS analysis data also confirm that the particles are Pd-rich intermetallics (Figure 5h). It is necessary to note that due to the small size of the particles compared to the region of interaction between the beam and the sample, the contribution of the matrix to the EDS spectrum is very large. However, the amount of palladium in the particles (2.49 wt%) is several times higher than the average concentration of palladium in the matrix (0.88 wt%). The particles are distributed inhomogeneously, and there are few of them. Due to the dispersity and small amount in the structure, they were not revealed by phase analysis.

**Figure 6:**
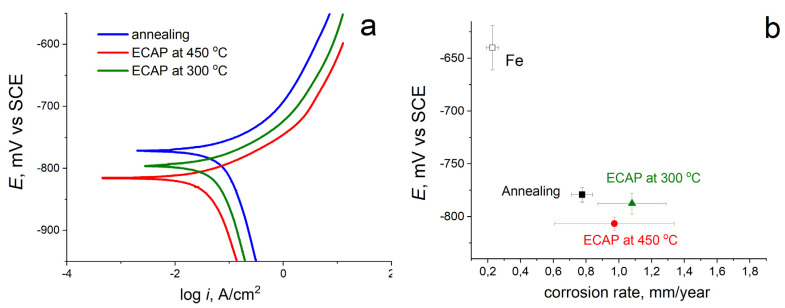
PDP curves in physiological saline solution at scan rate of 1 mV/s (**a**) and E vs. C_R_ (**b**) of Fe–Mn–Pd alloy in different states and technically pure iron for comparison.

The susceptibility of Fe–Mn–Pd alloy samples to corrosion was investigated in the course of electrochemical studies using the potentiodynamic polarization method (Figure 6). Figure 6a shows typical potentiodynamic curves for samples of Fe–Mn–Pd alloy in the initially annealed state and after ECAP at two deformation temperatures of 300 °C and 450 °C, in comparison with commercially pure iron. An analysis of the curves showed that after SPD by the ECAP method, the corrosion potential (*E_corr_*) of the alloys shifts to the negative region both relative to the corrosion potential of commercially pure iron (*E_corr_* = −623 ± m4 V) and relative to the initially annealed state (*E_corr_* = −779 ± 7 mV) (Figure 6, Table 4). When compared with the initially annealed state, *E_corr_* after ECAP at a temperature of 300 °C decreases slightly, within the error (up to *E_corr_* = −788 ± 9 mV) by ~9 mV, but after ECAP at a temperature of 450 °C, the corrosion potential decreases by ~28 mV. Apparently, this decrease occurs due to a larger amount of the second phase (IMP) in the alloy structure.

Despite the fact that precipitation of intermetallic compounds after ECAP at 300 °C is practically not visible, and after ECAP at 450 °C their presence is more noticeable, the average corrosion rates of the alloy after deformation in two modes at temperatures of 300 °C and 450 °C are almost the same and are equal to 1.08 ± 0.2 and 0.97 ± 0.36 mm/y, respectively (Figure 6b). The observed standard deviation for the corrosion rate of the alloy after ECAP at both temperatures is very large (Table 4).

**Figure 7:**
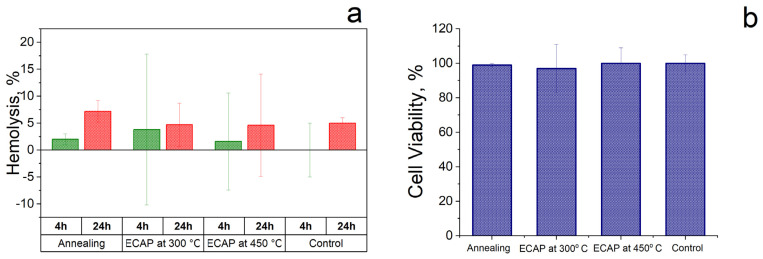
Study of the biocompatibility of Fe–Mn–Pd alloys in the initial state and after ECAP treatment by assessing the change in hemolysis (**a**) and cell viability (**b**) in comparison with the control.

To assess the effect of ECAP at temperatures of 300 °C and 450 °C on the biological activity of the Fe–Mn–Pd alloy, hemolysis and the level of viability of blood cells after incubation with the alloy samples were investigated. The level of hemolysis was measured after 4 and 24 h, and cytotoxicity after 24 h of incubation of samples with cells (Figure 7). The obtained results showed that the alloy samples after ECAP at different deformation temperatures did not induce significant changes in hemolysis and cell viability in comparison with the activity of the alloy samples in the initial state. The results obtained after incubation of cells with alloy samples in three states were compared with the control, where the cells were incubated under similar conditions but without contact with the alloy samples. Since no significant increase in hemolysis or decrease in the survival of blood cells was observed in the presence of all the studied samples, it can be concluded that the Fe–Mn–Pd alloy is biocompatible in the initially annealed state and after ECAP at two deformation temperatures of 300 °C and 450 °C.

**Figure 8:**
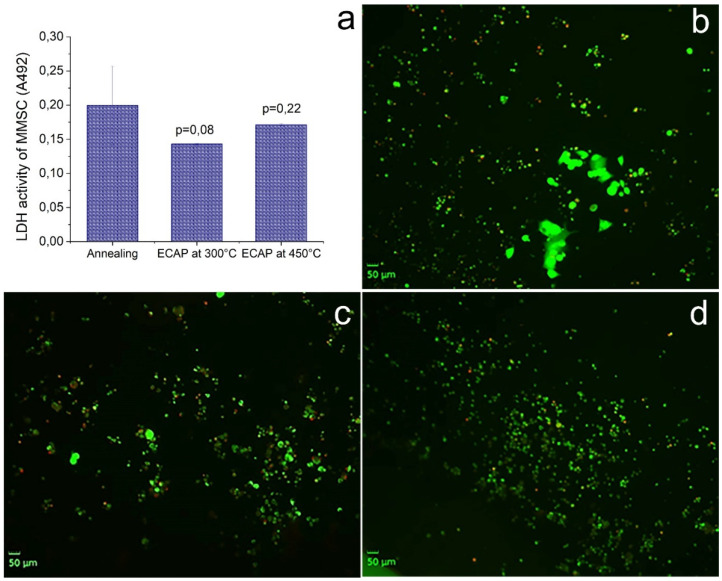
Cells colonization of the surface of Fe–Mn–Pd alloy samples in the initial state and after ECAP in two modes. LDH activity of MMSC (**a**) and fluorescence microscopy of MMSC colonized the surface of samples: annealed (**b**); ECAP-treated at 300 °C (**c**); ECAP-treated at 450 °C (**d**). Cells with a compromised membrane that are considered to be dead or dying were stained red, whereas live cells with intact membranes were stained green.

Along with biocompatibility, in the course of the described study, the colonization of the surface of the samples by cells was investigated. MMSCs were used as a model due to their osteogenic potential. It was found that all samples induced cell adhesion and colonization (Figure 8). At the same time, ECAP treatment did not lead to a significant change in the activity of the cell culture in comparison with the alloy in the initial state. The use of live/dead staining showed no cell death after adhesion to the surface of all studied alloy samples. Thus, it can be argued that ECAP and the corresponding change in the structure did not induce a decrease in the intensity of adhesion and colonization by cells with an osteogenic potential of the surface of the Fe–Mn–Pd alloy samples.

## 4. Discussion

In this work, the effect of SPD by the ECAP method at deformation temperatures of 300 °C and 450 °C on the structure and properties of a biodegradable Fe–Mn–Pd alloy for the manufacturing of medical devices was studied. As a part of the study, structure formation, changes in the phase composition, mechanical properties, corrosion resistance and biocompatibility were investigated.

The ECAP modes were selected according to the following design requirements. First, to obtain an ultrafine-grained structure with a significant volume of boundaries of structural elements, the maximum number of ECAP passes was used. Secondly, to obtain a fully austenitic structure, ECAP was carried out at elevated temperatures, taking into account the temperatures of the beginning and end of martensitic transformation for a binary Fe–Mn alloy with the same Mn content [36] and the authors’ own experience in obtaining a fully austenitic structure in metastable corrosion-resistant steels after SPD [37]. As a result, an austenitic UFG structure (Figure 2, Figure 4 and Figure 5) with a high dislocation density confirmed by X-ray line profile analysis (Table 2) was produced.

In order to choose the deformation temperature, the possibility of precipitation of dispersed intermetallic compounds during ECAP in Fe–Mn–Pd alloys at deformation temperatures below the precipitation temperature of IMP during annealing [16,17] was taken into account. It is important to note that SPD usually leads to a shift in the precipitation of particles towards lower temperatures [38]. A finer and more uniformly distributed IMP released during deformation could contribute not only to a greater rate but also to the uniformity of degradation. Unfortunately, the resulting structure had a high degree of inhomogeneity (Figure 4 and Figure 5). Some areas were twins of high density in austenite. In the areas of the subgrain structure, the localization of IMP particles of Mn_2_Pd_3_ with a size of 40 nm (Figure 4g and Figure 5g) occurred. Such heterogeneity of the structural-phase state led to a significant scatter in the values of the corrosion rate for ECAP at temperatures of 300 °C and 450 °C equal to 1.08 ± 0.2 and 0.97 ± 0.36 mm/y, respectively (Figure 6b, Table 4). A significant increase in the corrosion rate due to areas with IMP particles was offset by a decrease in corrosion due to twinning in austenite, as shown by recent studies [39]. Nevertheless, sufficiently high corrosion rates were obtained due to the presence of the UFG structure and IMP particles, and a larger number of IMP particles released during ECAP at a temperature of 450 °C was compensated by a higher dislocation density in samples after ECAP at 300 °C.

In addition, the results of the study of hemolytic activity and cytotoxicity led to the conclusion about the biocompatibility of the alloy, regardless of the processing mode used (Figure 7). It is important to note that a high level of mechanical properties (Figure 3, Table 3) was due to the austenitic UFG structure. It is well known that ECAP significantly improves the strength characteristics of iron and alloys based on it [19,22,23,24,25,26,27,28,29,40,41]. An increase in the specific strength of the alloys under study by the ECAP method would make it possible to reduce the size of the implanted product and, as a result, reduce the toxic effect of a foreign body in the human organism and the time of its degradation.

In the course of this work, ECAP proved to be an effective tool for regulating the structural-phase state, which is a determining factor in obtaining the required properties for specific operating conditions of medical devices made of biodegradable material. Moreover, it was shown that the treatment of the alloy in this mode does not change its cytoconductive properties, contributing to the colonization of the sample surface by cells with osteogenic potential. It is expected that this feature will favorably distinguish products from the developed alloy from titanium implants for compensating bone defects due to osteoconduction, covering by periosteum and the formation of effective osteosynthesis.

## 5. Conclusions

It was found that ECAP at temperatures of 300 °C and 450 °C leads to the formation of an ultrafine-grained austenitic partially twinned structure in the Fe–Mn–Pd alloy and the precipitation of particles of the Mn_2_Pd_3_ intermetallic phase.

The structure obtained after ECAP determined a high level of strength characteristics (σ*_UTS_* in the range from 1545 to 1669 MPa, σ*_YS_* in the range from 1488 to 1577 MPa) with a decrease in plasticity (ε in the range from 8.5 to 4%).

ECAP at temperatures of 300 °C and 450 °C increases the susceptibility of Fe–Mn–Pd alloy to corrosion by increasing corrosion rate up to 1.08 ± 0.2 and 0.97 ± 0.36 mm/y, respectively, due to structure refinement, increase in boundary density structural elements and precipitation of particles of the intermetallic phase Mn_2_Pd_3_.

It was found that samples of Fe–Mn–Pd alloy had no cytopathogenic effect on blood cells. The results of the study of hemolysis and cytotoxicity led to the conclusion about the biocompatibility of the alloy, regardless of the processing mode used.

It was found that all the studied samples of the Fe–Mn–Pd alloy after ECAP stimulated cell adhesion and colonization, while the treatment mode did not affect the intensity of bioactivity.

## Figures and Tables

**Table 1:** Chemical composition.

Alloy	Mn, %	Pd, %	Si, %	Cu, %	С, %	P, %	S, %	Fe, %
	in wt.%							
Fe	0.24	-	0.25	0.21	0.010	0.015	0.007	Bal.
Fe–Mn–Pd	22.20	0.88	0.78	0.38	0.01	0.013	0.005	Bal.

**Table 2:** Results of the X-ray line profile analysis of the Fe–Mn–Pd alloy after various processing.

Processing	Space Group	Phase	a, Å	Content, wt.%	Dislocation Density ρ, cm^−2^
Forging, at 1100 °С	225: Fm−3m	γ	3.623(9)	100.0(9)	-
Annealing, at 1250 °С (12 h)			3.647(1)	100.0(14)	-
ECAP, at 300 °С			3.625(7)	100.0(10)	5·10^11^
ECAP, at 450 °С			3.625	100.0(10)	1·10^11^

**Table 3:** Mechanical test results of the Fe–Mn–Pd alloy after various processing (annealed Fe for comparison).

№	Processing	σ*_UTS_* ^1^, MPa	σ*_YS_* ^2^, MPa	ε ^3^, %
1.	Forging, at 1100 °С	824	339	84.5
2.	Annealing, at 1250 °С (12 h)	643	280	65.5
3.	ECAP at 300 °C	1545	1488	8.5
4.	ECAP at 450 °C	1669	1577	4
5.	annealed Fe	403	324.5	40.6

^1^ ultimate tensile strength; ^2^ yield strength; ^3^ total elongation.

**Table 4:** Potentiodynamic polarization parameters of Fe–Mn–Pd alloy in physiological saline solution.

	Processing	E*_corr_* ^1^, mV	*i_corr_* ^2^*,* µmA/sm^2^	C_R_ ^3^, mm/y
Fe	Annealing for comparison	−623 ± 4	19.2 ± 2.4	0.23 ± 0.04
Fe–Mn–Pd	Annealing, at 1250 °С (12 h)	−779 ± 7	66.5 ± 5.5	0.78 ± 0.06
	ECAP at 300 °C	−788 ± 9	91.7 ± 18.3	1.08 ± 0.20
	ECAP at 450 °C	−807 ± 6	59.9 ± 47.7	0.97 ± 0.04

^1^ corrosion potential; ^2^ corrosion current density; ^3^ corrosion rate.

## Data Availability

All the data required to reproduce these experiments are present in the article.
